# Investigation of the Flexural and Charpy Impact Properties of Polymer Composites Reinforced with Tururi (*Manicaria saccifera)* Fibrous Fabric

**DOI:** 10.3390/polym17040466

**Published:** 2025-02-11

**Authors:** Avener Gleidson Andrade Santos, Damares da Cruz Barbosa Nascimento, Felipe Perissé Duarte Lopes, Noan Tonini Simonassi, Sérgio Neves Monteiro, Alisson Clay Rios da Silva, Verônica Scarpini Candido

**Affiliations:** 1 Materials Science and Engineering Program, Federal University of Pará—UFPA, Ananindeua 67130-660, Brazil; eng.avenersantos@gmail.com (A.G.A.S.); damares.barbosa62@gmail.com (D.d.C.B.N.); alissonrios@ufpa.br (A.C.R.d.S.); 2 Materials Science Program, North Fluminense State University—UENF, Campos dos Goytacazes 28013-602, Brazil; perisse@uenf.br (F.P.D.L.); noansimonassi@uenf.br (N.T.S.); 3 Materials Science Program, Military Institute of Engineering—IME, Rio de Janeiro 22290-270, Brazil; snevesmonteiro@gmail.com

**Keywords:** tururi fabric, characterization, mechanical properties

## Abstract

The search for new natural, sustainable, economical and biodegradable reinforcements for composite materials has increased in recent years, highlighting the importance of fibers from the natural environment. This work evaluates the use of tururi fibrous fabric as a reinforcement in a polymer matrix, using Fourier transform infrared spectroscopy, X-ray diffraction, thermogravimetry and scanning electron microscopy. The mechanical and fractographic performance of composites reinforced with 2.5, 5.0 and 7.5% mass fraction of tururi in a polyester matrix is also investigated. The FTIR and XRD results identified groups characteristic of natural fibers and the presence of elemental constituents such as cellulose, hemicellulose and lignin. Thermogravimetry indicated good thermal stability near 246 °C. The morphology of the fibrous fabric is irregular and formed by tangles of threads. The mechanical behavior of the composites in bending revealed a variation in stress with the increase in the percentage of fabric in the matrix, explained by defects and failures due to low interfacial adhesion between the phases. Impact tests indicated that increasing the percentage of fabric in the matrix improves impact energy absorption, reflecting better adhesion and load distribution. Thus, the development of this natural composite is promising for applications in green and sustainable products.

## 1. Introduction

Sustainable development is a determining environmental issue in the design of new materials. In this context, composite materials have been attracting attention on a global scale, as they have interesting environmental and mechanical requirements for various applications [1,2,3]. These materials are used on a large industrial scale due to their high resistance to stress and low specific mass. Industries such as aerospace, automotive, shipbuilding, packaging, and medicine and biomedicine use composite materials as a basis for the development of high- and low-intensity activities [4,5,6,7,8].

Increasing concerns about climate change and excessive carbon dioxide emissions have driven the development of sustainable, biodegradable and less environmentally aggressive materials [9]. As a result, the use of natural inputs from environmental reutilization, most notably natural fibers as reinforcements in polymer matrix composites, has leveraged the technical–scientific field in the manufacture of new products. The use of natural reinforcements has a number of advantages, such as low density, low purchase price, biodegradability and non-toxicity, as well as great environmental availability [7,10,11].

Lignocellulosic fibers from large and small plants have cellulose, hemicellulose, pectin and lignin as elementary components, in varying proportions [12,13,14,15]. For this reason, the use of environmentally recyclable natural inputs, particularly natural fibers as reinforcements in polymer matrix composites, is driving innovation in the technical–scientific field, leading to the manufacture of new products. The use of natural reinforcements has a number of advantages, such as low density, low purchase value, biodegradability and non-toxicity, as well as great environmental availability [7,10,11,16,17,18].

The main properties of composite materials reinforced with natural fibers are high mechanical strength due to the presence of cellulosic material, low density, high rigidity, low abrasiveness, wear resistance and a lower specific weight [19,20,21,22,23,24]. In addition, the arrangement and characteristics of the fibers, whether they are continuous or in the form of yarns or mats, make the composite material even more attractive for various industrial applications, with a view to replacing petroleum-derived fibers. Studies have been carried out to understand how the insertion of fabrics made from natural and synthetic fibers affects the properties of polymer matrix composite materials [25,26].

Oliveira et al. (2020) [27] developed polybenzozaxine composites reinforced with tururi fabric and observed a bending elastic modulus of 2 GPa and elevated glass transition temperatures. Ng et al. (2022) [28] observed that the manufacture of fabrics using natural and synthetic pineapple and glass fibers, respectively, results in lightweight composites with good mechanical properties that are environmentally interesting for application in the transport sector. In another study, Ng et al. (2024) [29] observed that the constitution of a hybrid fabric of ramie and pineapple fibers oriented in different directions shows high resistance to impact, traction and bending, due to the easy distribution of the load along the layers. In addition, they explain that hybrid fabrics have great potential for use, due to the environmentally friendly aspect of the material. Monteiro et al. [30] characterized tururi fabric and found tensile strength values of 17.6 MPa, as well as functional groups corresponding to cellulose, hemicellulose and lignin. Porras et al. [31] also characterized tururi fabric for use as a reinforcement in composites and concluded that cellulose and lignin were the main constituents in higher amounts, and that the fabric surface was coated with waxes and fats, suggesting that a chemical treatment could promote the removal of these compounds and improve interfacial adhesion. Midani et al. [32] studied the effects of the structural parameters of tururi fabric on the impact properties of multilayer composites and observed that increasing the number of layers led to a reduction in impact absorption capacity, with failure occurring due to matrix delamination, indicating lower fabric wettability by the resin.

On the scene of discoveries of new reinforcements for composite materials is tururi fabric. This fabric is extracted from the Ubuçu palm (*Manicaria saccifera*), a tree common in Latin America and used by native communities for a variety of applications. Straw and candles are made from the leaves, while drinks and other medicinal remedies are produced from the fruit, as well as hats and fishing accessories. The bracts form sacs that give rise to a fibrous fabric, which can be obtained in the form of an involucre [30,31]. The use of a natural fabric covers three dimensions (economic, social and sustainable development), requirements that make it interesting for use as a reinforcement. Although it is a fabric that has already been used as a reinforcement in composite materials [26,27,30,32] for the first time, the mechanical and dynamic mechanical behavior, as well as the Charpy impact energy, of polyester matrix composites reinforced with tururi fabric at mass percentages of 2.5, 5.0 and 7.5% are investigated, making it a relevant study in the field of composites reinforced with natural fiber fabrics, thus making it more attractive for the manufacture of new materials. Additionally, although the literature [26,27,30,32] provides relevant information on the fabric and composites, none of them investigated the reinforcement potential of tururi fabric in a polyester matrix, making the present study important for elucidating issues such as mechanical behavior as a function of temperature, the influence of fabric addition on the glass transition temperature of the polymer, and the absorbed impact energy. In this context, the objective of this work is to evaluate, through a series of analyses and tests, the potential use of a tururi fibrous fabric reinforcement for polyester polymer matrix composites.

## 2. Materials and Methods

**Figure 1:**
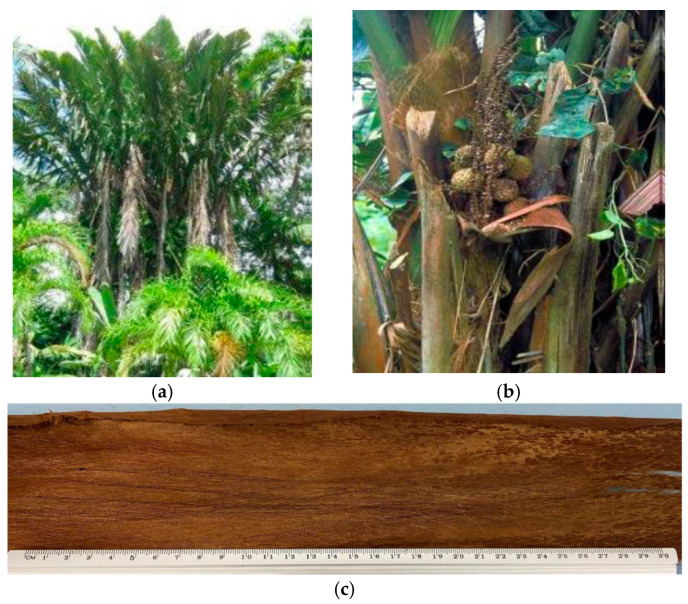
Ubuçu plant (**a**) [33], fabric extraction site [33] (**b**) and tururi fabric (**c**).

The tururi fibrous fabric was obtained commercially at the Ver-o-Peso market in Belém-PA, Brazil. The material was naturally obtained, without any previous chemical treatment. Figure 1 shows the ubuçu plant, the region where it was extracted and the fabric used.

The matrix used was a medium-viscosity isophthalic polyester resin and methyl ethyl ketone peroxide (MEK) hardening agent, supplied by Center Glass (Belém, PA, Brazil). The polyester resin was mixed with the hardening agent in a proportion of 1.5% by weight.

Table 1 shows the chemical composition of tururi fabric according to all TAPPI T203 and T222.

### 2.1. Composites Preparation

The composites were produced manually using the hand lay-up process, which consists of the dimensions of the ASTM D 790 [34] and ASTM D6110 [35] standards. The specimens were made using mass proportions of 2.5, 5.0 and 7.5% by weight of tururi fabric reinforcement, manually cut according to the specifications of each standard. The composites were produced manually using a silicone mold, where the tururi fabric was aligned longitudinally. After curing for 24 h, the specimens were demolded and sanded with 80 and 600 mesh grain sizes, followed by flexural and impact tests.

### 2.2. X-Ray Diffraction (XRD)

The qualitative crystalline phases were obtained by XRD in a Proto Manufacturing, XRD Powder Diffraction System: 30 kV and 2 mA generator, Cu-Kα1 radiation, angular step of 0.0149°, 0.5 s time interval, 47 min scan and 2θ ranging from 5° to 60°. The crystallinity index was calculated using Equation (1) [36].

(1) $$\mathrm{CI}\%=\left(1-\frac{I_{1}}{I_{2}}\right)\times 100$$

where the following are defined:

CI: crystallinity index; $I_{1}$: maximum intensity associated with the crystalline plane of cellulose; $I_{2}$: maximum intensity associated with the amorphous plane of cellulose.

#### Microfibril Angle (MFA)

The microfibrillar angle for tururi fabric was calculated using the Origin^®^ software program, based on the method described by Cave [37], considering the cellulose peak (0 0 2). Based on the relationship between the Gauss curve and the first- and second-order curves, the “T” parameter is obtained and then applied to the following expression:

(2) $$\mathrm{MFA}=-12.19\;\times \;T^{3}+113.67\;\times \;T^{2}-\;348.40\;\times \;T+358.09\;$$

### 2.3. Thermogravimetric Analysis (TGA)

Thermogravimetric analysis was carried out in a NETZSCH (Berlin, Germany) model STA 449 F3 analyzer. The atmosphere used was nitrogen a flow rate of 50 mL/min, a heating rate of 10 °C/min, and a temperature range from 25 to 500 °C.

### 2.4. Fourier Transform Infrared Spectroscopy (FTIR)

The FTIR of the fabric was carried out using BRUKER equipment, model VERTEX 70V, using an infrared range of 4000–400 cm^−1^. The FTIR analysis for the composites was carried out on equipment produced by Thermo Scientific, model NICOLET 6700 using an infrared range of 4000–500 cm^−1^.

### 2.5. SEM Analysis

A morphological analysis of the tururi fabric and fractographic analysis of the composites were carried out using a model Mira3 FEG 250 TESCAN microscope (Brno, Czech republic) operating with secondary electrons at 5 KV and a working distance ranging from 25 to 10 mm.

### 2.6. Mechanical Properties Tururi Fabric

The tururi fabric samples were tested according to the ASTM D5035, using an iM50 Universal Electromechanical Machine (Mogi das Cruzes, SP, Brazil), using a 5 kN load cell at a tensile and bending speed of 10 mm/min. Twenty specimens cut by hand according to the dimensions of the standard used were tested.

### 2.7. DMA

The DMA test was carried out using the single-cantilever configuration. The test was carried out on a TA instrument Q800 V21.3, at a temperature rate of 20 to 200 °C with heating of 3 °C per minute and frequency 1 Hz, in a nitrogen atmosphere. The dimensions used were 12 × 46 × 3 mm.

### 2.8. Flexure Test

The flexure test was carried out on EMIC electromechanical universal equipment model (DL500), with a 5 KN load cell and a loading rate of 2 mm/min. The test was carried out in compliance with ASTM D790–17 [34].

### 2.9. Charpy Impact Test

The Charpy impact test was carried out on instrumented pendulum equipment from PANTEC, model XC-50, 1× 200 V × 60 Hz with a pendulum of 0.7 J. The specimens were produced with prismatic dimensions and a 2.54 mm deep notch. The test was carried out in accordance with ASTM D6110 [35].

### 2.10. Statistical Analysis

A statistical validation of the data was carried out using the analysis of variance (ANOVA) tool, with a 95% confidence interval (*p* < 0.05). Mean values were compared using the Tukey test.

## 3. Results

### 3.1. Characterization of Tururi Fabric

#### 3.1.1. XRD Analysis

**Figure 2:**
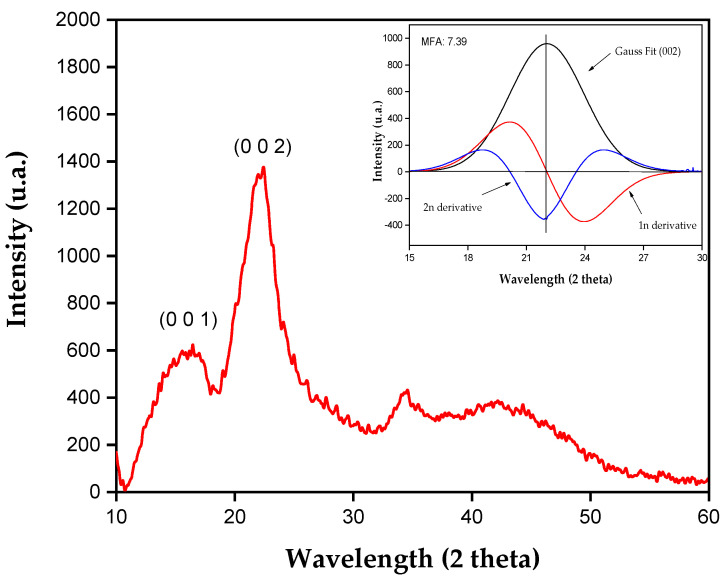
XRD pattern of the tururi fabric.

Figure 2 shows the x-ray diffractogram obtained for tururi fabric.

The XRD analysis shows halos at angles of 16.23° and 22.32°, characteristic of the 1 0 1 and 0 0 2 planes, respectively. The peaks at 16.23° and 22.32° indicate the presence of amorphous constituents such as hemicellulose, lignin and pectins, in addition to crystalline cellulose. These results are similar to those found for natural fibers such as guaruman [38,39], ubim [40] and periquiteira [41].

The crystallinity index of the tururi fabric was 46.67%.

Table 2 compares the crystallinity index results of different natural fibers.

Although the tururi fabric has a lower crystallinity index, it still outperforms fibers such as pineapple, *Citrullus lanatus* and *Cereus hildmannianus*, which have exhibited even lower indices [48,50,52].

Natural fibers have compatible microfibril structural arrangements, which determine the microfibril angle (MFA). Fibers with a low MFA, above 0°, tend to be stronger and stiffer, while fibers with a high MFA, above 90°, are more flexible and susceptible to fracture [45].

The microfibrillar angle of the tururi fabric, calculated from the peak intensity of 22.32° [48], was 7.39°. This value is close to the value obtained for other lignocellulosic fibers, such coconut palm fiber, *Eurcraea foetida*, Ubim and periquiteira [40,41,53,54]. According to JUNIO et al. [41], low MFA values indicate good mechanical properties, making these fibers suitable for reinforcements.

The results show that tururi fabric has structural characteristics typical of natural fibers. The presence of these constituents reinforces the potential use of the fabric in polymer matrix composite materials.

#### 3.1.2. TGA

**Figure 3:**
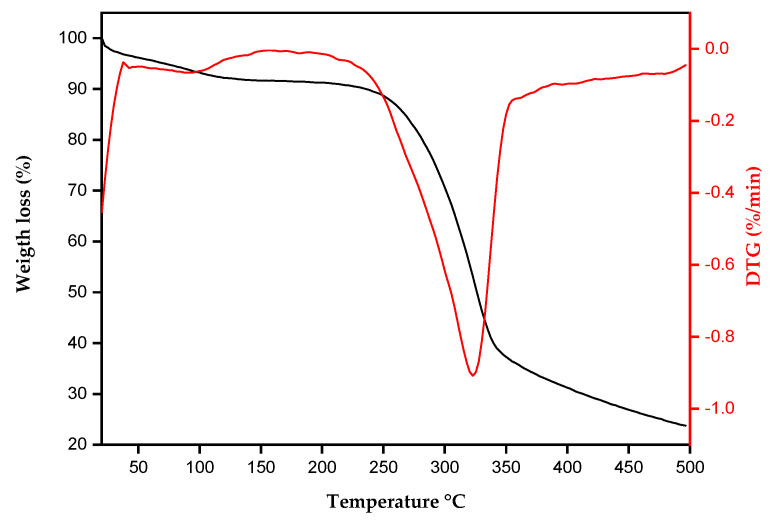
TG and DTG for tururi fabric.

The thermogravimetric curve (TG) and the thermogravimetric derivative (DTG) for tururi fibrous fabric are shown in Figure 3.

The thermogravimetric (TG) curve shows three distinct mass loss events. The first occurs at around 102 °C, indicating the loss of residual moisture, as reported by Dalmis et al. [55] and Khan et al. [56]. The second event occurs at approximately 247 °C, associated with the initial thermal degradation of hemicellulose and pectins present in lignocellulosic fibers, in addition to the release of all structural water [57,58]. The third and final thermal degradation event occurs at around 334 °C, corresponding to the beginning of the degradation of cellulose and other organic components [59]. This process involves the depolymerization, decarboxylation and decomposition of the glucose units present in the structure of lignocellulosic fibers [60,61]. The thermogram shows that tururi fibrous fabric maintains good thermal stability up to approximately 246 °C. Above this temperature, the thermal degradation of the fabric begins. The results suggest that tururi fabric has adequate thermal stability, which makes it promising for applications in polymeric matrices.

#### 3.1.3. FTIR Analysis

**Figure 4:**
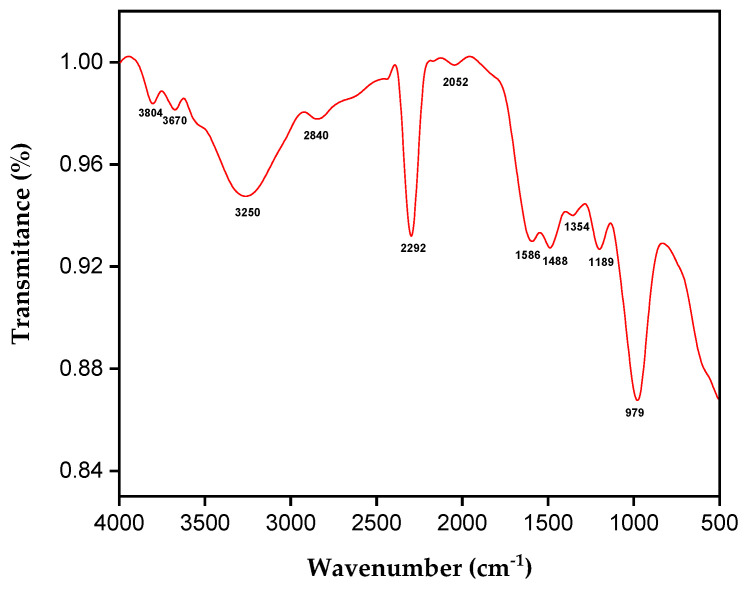
FTIR of tururi fabric.

Figure 4 shows the spectrum for tururi fibrous fabric.

FTIR is an important characterization technique for identifying the functional groups or absorption bands of constituents. Between 3600 and 3100 cm^−1^, two vibrational bands are observed that can be attributed to the stretching of the hydroxyl group (OH-) [62,63]. At 2840 cm^−1^, the vibrational stretching of the CH and CH_2_ bonds, constituents of cellulose and hemicellulose, is observed [64]. At 2355 cm^−1^, there is stretching of the C=C bonds and the presence of waxes [41,65]. The absorption band at 2052 cm^−1^ corresponds to the stretching of the C≡N and C≡C bonds [66]. At 1586 cm^−1^, the stretching of the C-C bonds of the aromatic lignin ring is observed [31]. The peak observed at 1488 cm^−1^ corresponds to the C-H bond bending frequency of cellulose [62]. At 1354 cm^−1^, there is stretching of the acetyl group of lignin [31]. The band at 1189 cm^−1^ corresponds to the stretching of the C-O-C group of the lignin and hemicellulose constituents [51]. At 979 cm^−1^, it is associated with the crystalline cellulose constituent [60]. Tururi fabric shows absorption bands characteristic of natural fibers.

#### 3.1.4. SEM Analysis of Tururi Fabric

**Figure 5:**
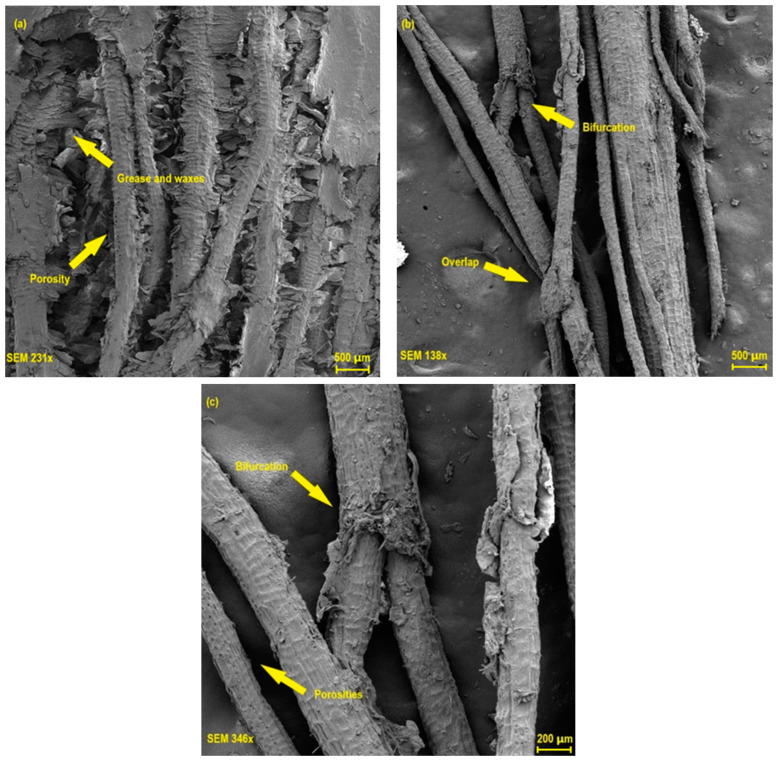
Scanning electron microscopy obtained for the tururi fabric. (**a**) tururi fabric yarns oriented on different surfaces, 231×, (**b**) tuturi fabric with magnification of 138× and (**c**) tuturi fabric with magnification of 348×.

The morphology of tururi fabric was analyzed using scanning electron microscopy, as shown in Figure 5.

Figure 5a shows that the tururi fibrous fabric is structured by yarns oriented on different surfaces, as well as having heterogeneous and irregular surfaces formed by roughness, pores and organic matter such as grease and wax [64,67]. Although the presence of pores and imperfections limits the mechanical performance of the fabric, these aspects can contribute positively to better anchoring and interfacial adhesion between the fabric and the polymer matrix, favoring the formation of more resistant and rigid green materials [27,49]. Oliveira et al. [68] and Porras et al. [31] also observed similar behavior when investigating the properties of tururi fabric. In Figure 5b,c, areas can be observed composed of interlacing, bifurcations and overlapping of threads that present characteristics of the fabric. The bifurcations have a fibrous structure and help with load distribution, the breaking mode and the interaction between the matrix and the fabric. Karthik et al. [69] and Gao et al. [70] explained that the structural organization of yarns or fibers directly determines the properties of composite materials.

Tururi fabric has morphological characteristics similar to those of other natural fibers already investigated in the literature, such as *Vachellia farnesiana*, *Citrullus lanatus* and *Cortaderia selloana* [52,56,71].

#### 3.1.5. Mechanical Properties Tururi Fabric

**Figure 6:**
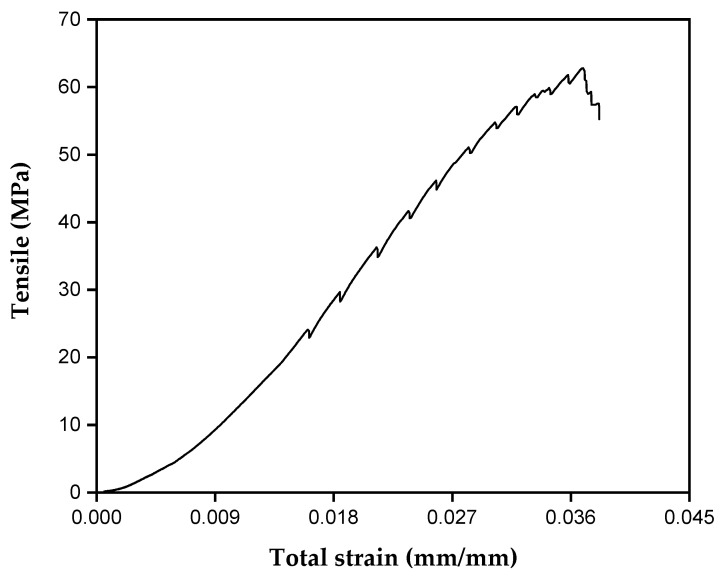
Tension versus strain curve of natural tururi fabric.

Figure 6 illustrates the tensile strength of the tururi fabric as a function of strain.

The tururi fabric had a maximum tensile strength peak of 60 MPa and an average Young’s modulus of 1.1 GPa. After reaching the point of maximum tension, the filaments of the fabric tend to fail in different directions, due to the redistribution of tensions and the breaking of the fibers. This characteristic is associated with the structural design of tururi fabric, which comprises natural fibers arranged in multiple directions or interlaced, as described by Porras et al. [31].

The results obtained for tururi fabric are superior to those of other natural fibers, such as *Corypha taliera* [66] and coconut [72]. However, studies carried out by Akter et al. (2025) [73] indicated that fabrics made from jute fibers have superior mechanical performance compared to tururi fabric. However, the properties of tururi fabric, combined with its environmental characteristics, reinforce its potential as a reinforcement material in sustainable composite applications.

### 3.2. Characterization of Composite Materials

#### 3.2.1. FTIR Composite Analysis

**Figure 7:**
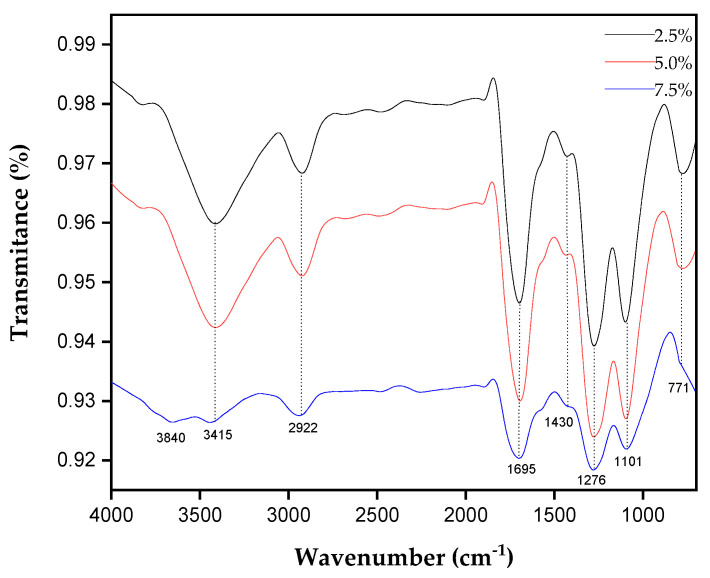
FTIR of composite materials reinforced with tururi fabric.

Figure 7 shows the spectrum obtained for the composites reinforced with tururi fabric with different percentages of incorporation.

The band observed at 3415 cm^−1^ is characteristic of the stretching of the hydroxyl cluster [2]. The band observed at 2922 cm^−1^ corresponds to the asymmetric stretching of the CH bond. At 1695 cm^−1^, an expressive peak is presented which is related to the grouping between the C=C bond of the carbonyl [2]. The band observed at 1430 cm^−1^ corresponds to the stretching of the bonds in the aromatic ring of lignin [74]. At 1276 cm^−1^, it refers to the stretching of the CO bonds of the acetyl ketone group [55]. At 771 cm^−1^, it corresponds to the stretching of the C=H bond [2,75]. As the percentage of the fabric increases, the transmission bands decrease, confirming that the percentage variation had an influence on the composition of the material.

#### 3.2.2. DMA of Composites

**Figure 8:**
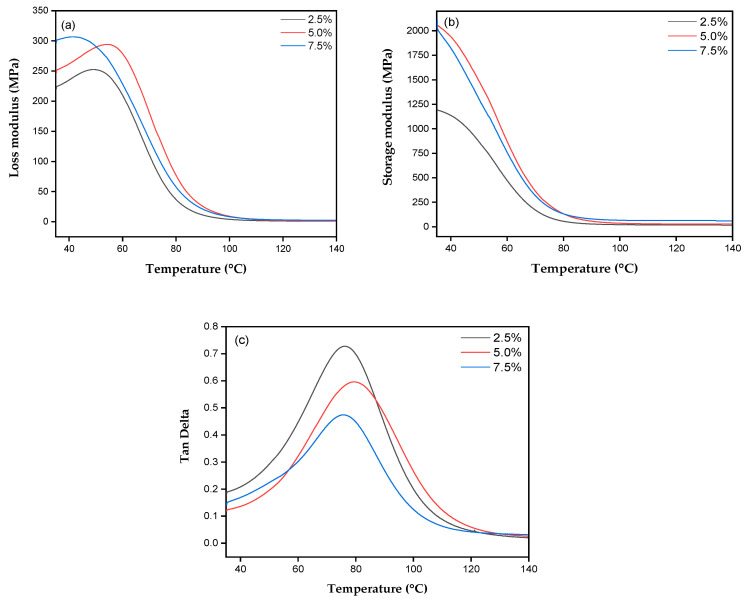
DMA curves of the composites of (**a**) loss modulus, (**b**) storage modulus and (**c**) tan delta.

Figure 8 shows the dynamic storage modulus (E′), tan delta (δ) and loss storage modulus (E″) for the composites with 2.5, 5.0 and 7.5% mass fraction of fabric in relation to temperature.

The DMA curves show that the composites reinforced with 2.5% fabric had an E′ equal to 1195 MPa, while the composites reinforced with 5.0 and 7.5% exhibit an E′ of 2077 and 2107 MPa, respectively. The increase in the percentage of fabric, as well as the increase in temperature, influenced the decrease in the storage modulus of the composites. These results suggest that increasing the percentage of reinforcement can provide greater rigidity and thermal and mechanical stability, as well as influencing movement in the vitreous transition region. In addition, the formation of bonds prevents the movement of the polymer chains, increasing the storage modulus. In this case, high stress is required to break the bonds between the interfaces and cause the material to rupture [76].

The loss of storage behavior shows that the 2.5% curve has a lower peak and a lower vitreous transition temperature. However, the composites with 5 and 7.5% reinforcement showed higher E″ peaks and a higher vitreous transition temperature compared to the composites with 2.5%. This indicates that the increase in reinforcement restricts the movement of the polymer chains due to the density of the cross-links, suggesting that there is an interaction between the fabric and the matrix [15].

The tan delta, which evaluates the material’s viscoelastic energy dissipation, showed that higher reinforcement percentages of 5 and 7.5% tururi fabric dissipated less energy during strain, resulting in a rigid and mechanically efficient material. This behavior suggests that increasing the reinforcement content contributes positively to the structure of the material, reducing the exclusive dependence on the polymer matrix for mechanical performance.

#### 3.2.3. Flexure Test

**Figure 9:**
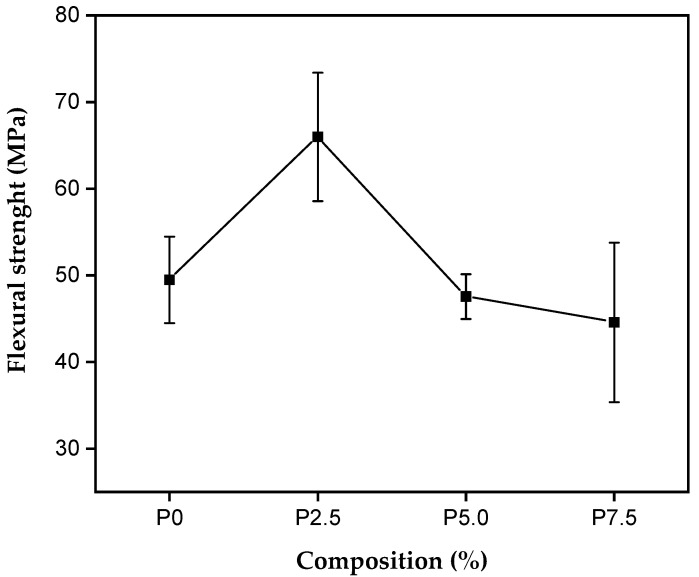
Flexural strength of composites reinforced with tururi fabrics.

The flexural strength of the polyester matrix composites reinforced with tururi fibrous fabric is shown in Figure 9.

Table 3 shows the flexural strength, modulus of elasticity and strain results for the composites reinforced with natural tururi fabric.

The composition with 2.5% reinforcement showed the best flexural strength results, with an average of 65 MPa. The compositions with 5.0% and 7.5% reinforcement showed lower results than the polyester matrix and the composition with 2.5%. The relative decrease in flexural strength may be associated with the presence of organic compounds in the fibrous fabric, which make it difficult to anchor the reinforcement to the matrix [77,78]. In addition, it is suggested that the presence of pores, microstructural damage and bubbles act as stress concentrators, reducing the mechanical strength of the composite material [79]. Karthik et al. [69] explain that conventional interfacial bonding between the fiber or fabric and the matrix results in low flexural strength.

**Figure 10:**
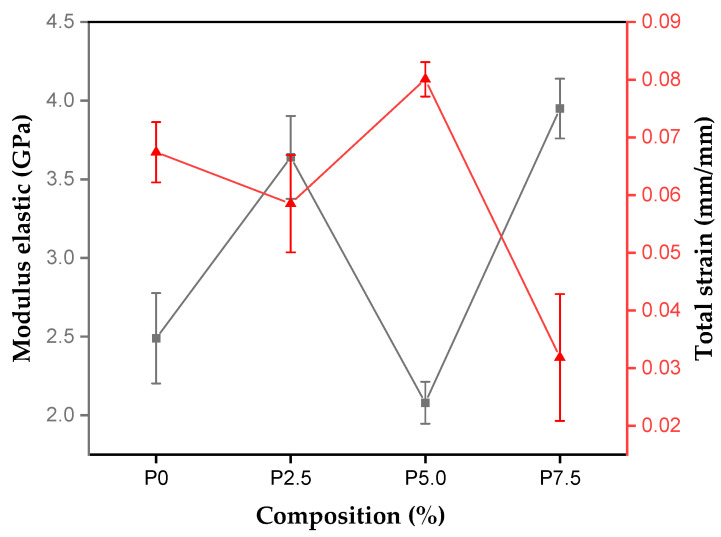
Modulus of elasticity and strain results for composites with tururi fabric.

Figure 10 shows the results of the modulus of elasticity and strain of the composites reinforced with tururi fabric.

The composites produced with 7.5% reinforcement had a higher modulus of elasticity, with an average of 3.95 GPa. The increase in modulus of elasticity is associated with the uniformity and homogeneity of the microfibrils of the tururi fabric, helping to distribute the loads throughout the matrix [80].

It should be noted that as the percentage of reinforcement increases, there is also an increase in the deformation of the material, resulting in an increase in flexural strength and modulus of elasticity. This behavior can be explained by the increase in the percentage of fabric and the increase in interfaces, which reduces the deformation capacity. The random arrangement of the yarns in the fabric reduces delaminations and increases the flexural strength of the material [81,82]. NG et al. [29] explain that, during bending tests, the top layer of the fabric tends to support compression loads, while the lower layers support the tension offered. In this way, the fabrics in the outer layers tend to support more bending loads than the other layers of the material.

**Figure 11:**
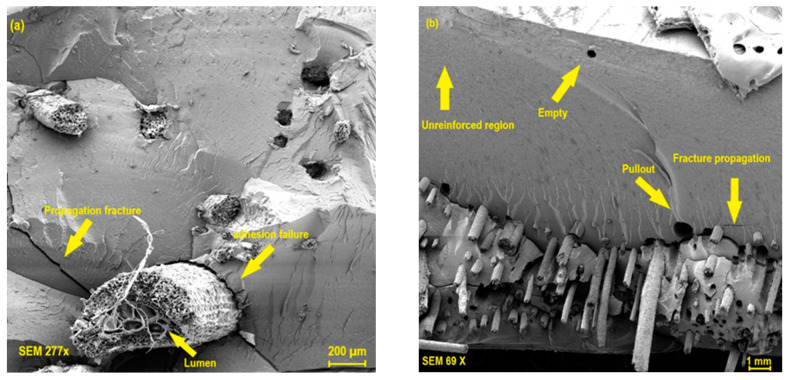
Scanning electron microscopy obtained for the composites in percentages of 2.5 (**a**) and 7.5% (**b**) in mass fraction of tururi fabric.

The morphology of the flexural fractures for the composites reinforced with 2.5% and 5.0% tururi fabric is shown in Figure 11a and Figure 11b, respectively.

It is possible to observe the presence of river marks which indicate that the fracture is of the brittle type for the composites with 2.5 and 5.0% fabric percentage. In addition, regions characteristic of the pull-out effect can be seen, as well as voids and pores, indicating that there was low adhesion between the fabric and the polymer matrix. This interaction results in a reduction in the flexural strength of the 2.5% and 5.0% compositions. Simonassi et al. [83] explains that the pull-out effect and the presence of voids are attributed to interfacial phenomena between the fiber and the polymer matrix, which tend to reduce the mechanical properties of composite materials [81]. Other characteristics associated with the hydrophobic characteristics between the matrix and the reinforcement can promote low interaction between the phases, causing flaws and defects along the length of the material [84]. In addition, when subjected to static loads or dynamic events, they lead to early rupture. These effects reduce the mechanical properties of composite materials reinforced with natural tururi fabric.

#### 3.2.4. Charpy Impact Test

**Figure 12:**
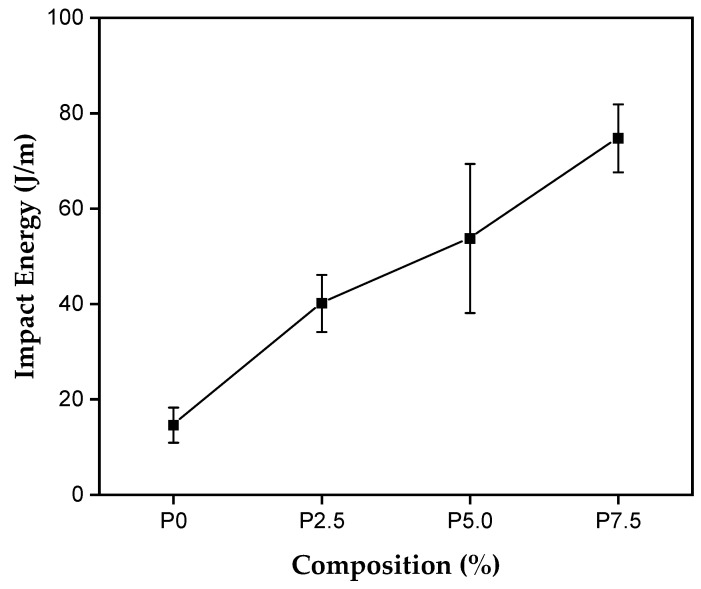
Impact energy for polyester matrix and composites reinforced with tururi fabric.

The results of the impact resistance test of the polymer matrix composites with different percentages of tururi fabric are shown in Figure 12.

Table 4 shows the Charpy impact energy results for the composites reinforced with natural tururi fabric.

The mean results show that the matrix and the 7.5% composition had around 14.61 J/m and 74.72 J/m, respectively. The composites produced with 7.5% showed an increase of 471.2% compared to the matrix. Therefore, the increase in impact resistance can be attributed to the increase in the percentage of reinforcement in the polymer matrix, resulting in an improvement in mechanical properties [85]. Pereira et al. [86], Pereira et al. [87] and Candido et al. [88] observed that the increase in impact energy absorption capacity is high when the percentage of reinforcement in the matrix is increased. From this, it can be said that the toughness resistance is increased by incorporating tururi fabric into the polyester matrix.

Fabrics made with natural or synthetic fibers oriented in the longitudinal and transverse directions have greater impact and load resistance due to the alignment of the fibers. In addition, fabrics, whether natural or synthetic in structure, tend to absorb more energy when compared to composites made with unidirectionally aligned fibers [89]. Chaves et al. [90] explain that since fibers are stiffer and stronger than the matrix, these properties result in stiffer and stronger composites, proportionally to the increase in the percentage of fibers.

The significant increase in impact energy is intrinsically linked to the characteristics and particularities of natural tururi fabric.

**Figure 13:**
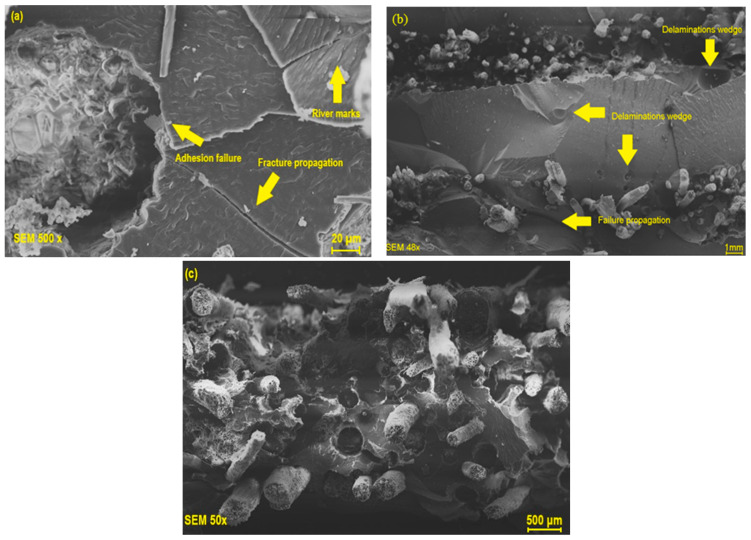
Micrographs of composites reinforced with 2.5% (**a**), 5.0% (**b**) and 7.5% (**c**) mass fraction of tururi fabric.

Micrographs of the fractures of composites subjected to the Charpy impact test are shown in Figure 13.

Figure 13a shows marks that appear as rivers, as well as the propagation of cracks, which indicate brittle fracture. Figure 13b shows the presence of voids and propagated cracks, which result from low energy dissipation along the matrix and reinforcement. Figure 13c reveals the presence of voids or areas marked by the pull-out effect, which manifests itself when the material is subjected to the transfer of impact energy [91]. Ng et al. [29] explains that the stacking and microstructural characteristics of the fabric can lead to separations, delaminations and the pull-out effect. These factors are related to the interfacial bonding between the phases, which may be insufficient to ensure that the material absorbs even more energy, resulting in the pull-out and rupture of the material when subjected to high loads [90,92].

In addition to the failure mechanisms, the structural organization of tururi fabric facilitates better adhesion with the polymer matrix and, consequently, a better distribution of stresses throughout the material. According to Gao et al. [70] and Karthik et al. [69], fabrics comprising multilayers of synthetic or natural fibers with randomly arranged yarns favor an increase in the energy absorption and impact resistance of the composite material. Furthermore, as the relative percentage of tururi reinforcement increases, there is a reduction in apparent defects.

The use of a natural fabric reinforces the development of lightweight and sustainable composite materials with exceptional properties for various applications.

#### 3.2.5. Statistical Analysis

Table 5 presents the analysis of variance for the mechanical characterization of composites reinforced with tururi fabric.

The ANOVA results for flexural strength indicate that the calculated F (8.629) is higher than the critical F (3.490). For deformation, the calculated F was (17.288) and the critical F was equal to (3.490). For the modulus of elasticity, the calculated F (28.951) was higher than the critical F (3.490). The impact resistance result showed a calculated F (22.099) greater than the critical F (3.490). Based on these results, the null hypothesis of equality between the means of the properties evaluated was rejected, with a 95% confidence level. After rejecting equality on the basis of the ANOVA result, Tukey’s test was applied to identify which means differed significantly in terms of the variation in the percentage of reinforcement in the polymer matrix.

Table 6 shows the results of Tukey’s test.

The minimum significant difference (msd) is a parameter that differentiates the averages obtained in the tests. For the properties of flexural strength, modulus of elasticity, deformation and impact resistance, the msd values were 14.810, 0.755, 0.022 and 24.280, respectively.

The results of Tukey’s test for flexural strength indicated statistical differences between the composites produced with 2.5% fabric and the other compositions. These data suggest that the composites with 2.5% fabric are less ductile but have greater strength. The differences observed in the modulus of elasticity averages confirm that adding more than 2.5% fabric significantly increases the rigidity of the material.

The Charpy impact resistance test results showed statistical differences. Composites with 5.0% and 7.5% tururi showed significant differences compared to composites with the lower percentages of 0% and 2.5%. These results indicate that increasing the percentage of fabric is associated with better impact properties.

Thus, incorporating tururi fabric into the polymer matrix tends to improve the properties of flexural strength, deformation, modulus of elasticity and impact resistance.

The characterization of tururi revealed that the fabric exhibited thermal stability up to approximately 250 °C, indicating that it can be used as a reinforcement agent for more thermally stable matrices. Furthermore, its use as a reinforcement agent in polymeric matrices showed low flexural strength values, as observed by [69,78], although these are still considered relatively high values for this property. Due to its energy absorption capacity upon impact and its visual appearance, it is suggested that the produced composite could be employed in the construction sector for the manufacturing of cladding panels and exposed bricks wall.

## 4. Conclusions

The characterizations of the tururi fabric and the composites revealed the following observations:

FT-IR indicated the presence of functional groups characteristic of the constituents present in tururi fabric, such as cellulose, hemicellulose and lignin. The thermogram showed the water loss from and degradation of the elemental constituents present in the fabric, as well as thermal stability at temperatures of up to 220 °C. From the XRD analysis, it was possible to identify the microfibrillar angle (7.39°) and the crystallinity index (46.67%). A morphological analysis of the tururi fabric revealed the presence of imperfections, flaws, tangles and irregular microstructural features in the fabric, as well as bifurcation regions. In the mechanical characterization by flexural test, the composites with 2.5% reinforcement showed a flexural strength and modulus of elasticity of 65 MPa and 3.76 GPa, respectively. A DMA indicated that increasing the percentage of fabric in the polymer matrix influenced the changes in vitreous temperature and the final properties of the composite material. The Charpy impact test indicated that the composition with 7.5% reinforcement showed an increase in energy absorption capacity of approximately 471% compared to the matrix. This result suggests that the energy absorption capacity increases with the incorporation of reinforcement. A fracture analysis of the composites indicated the presence of microstructural imperfections, such as porosity, voids and poor distribution of the reinforcement, attributed to the processing phase. The decrease in mechanical strength is intrinsically related to the morphological characteristics of the fabric. Compared to other studies using natural fibers as reinforcement, the new composite reinforced with fibrous fabric in a polyester polymer matrix shows great potential for applications subject to dynamic loads. Moreover, due to its good mechanical properties, tururi is an attractive alternative for use in composite materials because it is a completely natural fabric derived from a renewable natural resource.

## Figures and Tables

**Table 1:** Chemical analysis of tururi fabric.

	Cellulose (%)	Hemicellulose (%)	Lignin (%)	Reference
Tururi Fabric	74.1	12.0	31.1	[31]

**Table 2:** Comparative results of crystallinity indices (CIs) of different natural fibers and tururi fabric.

Fiber	CI (%)	References
Tururi Fabric	46.67	Present Work
Carnauba	86.90	[42]
Hemp	82.10	[43,44,45]
Jack Tree Fiber	86.00	[46]
Curauá	75.60	[47]
Pineapple	38.00	[48]
Ubim	83.00	[40]
Periquiteira	70.49	[41]
Buriti	63.00	[49]
*Cereus hildmannianus*	40.19	[50]
*Calamus manan*	48.28	[51]
*Citrullus lanatus*	33.33	[52]

**Table 3:** Flexural strength results for tururi/polyester composites.

Sample	Flexural Strength (MPa)	Modulus Elastic (GPa)	Strain (mm/mm)
P0	49.47 ± 4.99	2.49 ± 0.28	0.065 ± 0.005
P2.5	65.97 ± 7.41	3.64 ± 0.26	0.058 ± 0.008
P5.0	47.55 ± 2.58	2.08 ± 0.13	0.080 ± 0.003
P7.5	44.57 ± 9.20	3.95 ± 0.18	0.031 ± 0.011

**Table 4:** Charpy impact results for Tururi/polyester composites.

Sample	Charpy Impact Resistance (J/m)
P0	14.61 ± 3.69
P2.5	40.13 ± 5.60
P5.0	53.73 ± 15.63
P7.5	74.72 ± 7.13

**Table 5:** Analysis of variance for results.

**Flexural Strengh (MPa)**						
Source	Sum squares	Degree of freedom	Mean of squares	F (calculated)	*p*-value	F-critical
Between the groups	110,683.7	3	368.945	8.629	0.002	3.490
Inside the groups	513.064	12	42.755			
Total	161,990.2	15				
**Elastic Modulus (GPa)**						
Source	Sum squares	Degree of freedom	Mean of squares	F (calculated)	*p*-value	F-critical
Between the groups	9.652	3	3.217	28.951	8.88 × 10^−6^	3.490
Inside the groups	1.333	12	0.111			
Total	10.986	15				
**Total Strain (mm/mm)**						
Source	Sum squares	Degree of freedom	Mean of squares	F (calculated)	*p*-value	F-critical
Between the groups	0.005	3	0.001	17.288	1.181 × 10^−4^	3.490
Inside the groups	0.001	12	9.916 × 10^−5^			
Total	0.006	15				
**Charpy Impact Resistance (J/m)**						
Source	Sum squares	Degree of freedom	Mean of squares	F (calculated)	*p*-value	F-critical
Between the groups	7618.892	3	2539.631	22.099	3.54 × 10^−5^	3.490
Inside the groups	1379.001	12	114.916			
Total	8997.893	15				

**Table 6:** Presents the results of the Tukey Test.

**Flexural Strength (m.s.d: 14.810)**				
**Sample**	**P0**	**P2.5**	**P5.0**	**P7.5**
**P0**	0	16.018 *	1.925	4.905
**P2.5**	16.501 *	0	18.427 *	21.407 *
**P5.0**	1.925	18.427 *	0	2.980
**P7.5**	4.905	21.407 *	2.980	0
**Elastic modulus (m.s.d: 0.755)**				
**Sample**	**P0**	**P2.5**	**P5.0**	**P7.5**
**P0**	0	1.155 *	0.405	1.462 *
**P2.5**	1.155 *	0	1.56 *	0.307
**P5.0**	0.405 *	1.56 *	0	1.867 *
**P7.5**	1.462 *	0.307	1.867 *	0
**Total Strain (m.s.d: 0.022)**				
**Sample**	**P0**	**P2.5**	**P5.0**	**P7.5**
**P0**	0	0.007	0.015	0.034 *
**P2.5**	0.088 *	0	0.022	0.027 *
**P5.0**	0.015 *	0.022	0	0.049 *
**P7.5**	0.034 *	0.027 *	0.049 *	0
**Impact Energy (m.s.d: 24.280)**				
**Sample**	**P0**	**P2.5**	**P5.0**	**P7.5**
**P0**	0	25.526 *	39.125 *	60.118 *
**P2.5**	25.526 *	0	13.598	34.591 *
**P5.0**	39.125	13.598	0	20.993
**P7.5**	60.118 *	34.591 *	20.993	0

* Statistically different values.

## Data Availability

The original contributions presented in this study are included in the article. Further inquiries can be directed to the corresponding author.
